# 6-(4-Amino­phen­yl)-2-meth­oxy-4-phenyl­nicotino­nitrile

**DOI:** 10.1107/S1600536813031437

**Published:** 2013-11-23

**Authors:** Thitipone Suwunwong, Suchada Chantrapromma, Ching Kheng Quah, Hoong-Kun Fun

**Affiliations:** a Department of Chemistry, Faculty of Science, Prince of Songkla University, Hat-Yai, Songkhla 90112, Thailand; b X-ray Crystallography Unit, School of Physics, Universiti Sains Malaysia, 11800 USM, Penang, Malaysia

## Abstract

In the structure of the title nicotino­nitrile derivative, C_19_H_15_N_3_O, the pyridine ring makes dihedral angles of 11.50 (7) and 43.36 (8)° with the 4-amino­phenyl and phenyl rings, respectively, and the dihedral angle between the phenyl rings is 36.28°. In the crystal, mol­ecules are linked by N—H⋯N hydrogen bonds into wave-like sheets parallel to (10-2). These sheets are stacked by π–π inter­actions between the 4-amino­phenyl rings of adjacent sheets, with centroid–centroid distances of 3.7499 (9) Å. C—H⋯π inter­actions are also present.

## Related literature

For the synthesis and applications of nicotino­nitrile derivatives, see: Al-Jaber *et al.* (2012 ▶); Brandt *et al.* (2010 ▶); El-Sayed *et al.* (2011 ▶); Ji *et al.* (2007 ▶); Kim *et al.* (2005 ▶); Koner *et al.* (2012 ▶); Raghukumar *et al.* (2003 ▶); Zhou *et al.* (2006 ▶). For bond-length data, see: Allen *et al.* (1987 ▶). For related structures, see: Chantrapromma *et al.* (2013 ▶); Suwunwong *et al.* (2012 ▶, 2013 ▶).
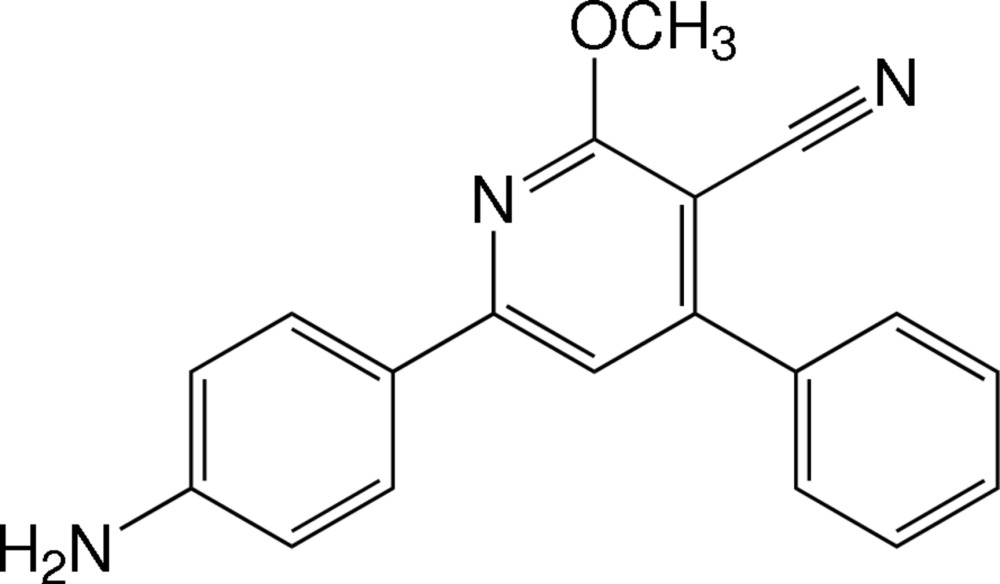


## Experimental

### 

#### Crystal data

C_19_H_15_N_3_O
*M* *_r_* = 301.34
Monoclinic, 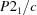

*a* = 10.9448 (12) Å
*b* = 18.960 (2) Å
*c* = 7.4738 (8) Å
β = 94.743 (2)°
*V* = 1545.6 (3) Å^3^
*Z* = 4
Mo *K*α radiation
μ = 0.08 mm^−1^
*T* = 100 K
0.56 × 0.17 × 0.06 mm

#### Data collection

Bruker APEX DUO CCD area-detector diffractometer
Absorption correction: multi-scan (*SADABS*; Bruker, 2009 ▶) *T*_min_ = 0.955, *T*_max_ = 0.995
14200 measured reflections
3696 independent reflections
2689 reflections with *I* > 2σ(*I*)
*R*_int_ = 0.051

#### Refinement

*R*[*F* ^2^ > 2σ(*F* ^2^)] = 0.046
*wR*(*F* ^2^) = 0.143
*S* = 1.07
3696 reflections
217 parameters
H atoms treated by a mixture of independent and constrained refinement
Δρ_max_ = 0.30 e Å^−3^
Δρ_min_ = −0.28 e Å^−3^

### 

Data collection: *APEX2* (Bruker, 2009 ▶); cell refinement: *SAINT* (Bruker, 2009 ▶); data reduction: *SAINT*; program(s) used to solve structure: *SHELXTL* (Sheldrick, 2008 ▶); program(s) used to refine structure: *SHELXTL*; molecular graphics: *SHELXTL*; software used to prepare material for publication: *SHELXTL*, *PLATON* (Spek, 2009 ▶), *Mercury* (Macrae *et al.*, 2006 ▶) and *publCIF* (Westrip, 2010 ▶).

## Supplementary Material

Crystal structure: contains datablock(s) global, I. DOI: 10.1107/S1600536813031437/sj5368sup1.cif

Structure factors: contains datablock(s) I. DOI: 10.1107/S1600536813031437/sj5368Isup2.hkl

Supplementary material file. DOI: 10.1107/S1600536813031437/sj5368Isup3.cml

Additional supplementary materials:  crystallographic information; 3D view; checkCIF report

## Figures and Tables

**Table 1:** Hydrogen-bond geometry (Å, °) *Cg*2 and *Cg*3 are the centroids of the C1–C6 and C10–C15 rings, respectively.

*D*—H⋯*A*	*D*—H	H⋯*A*	*D*⋯*A*	*D*—H⋯*A*
N1—H2*N*1⋯N3^i^	0.915 (19)	2.338 (19)	3.2416 (18)	169.5 (19)
N1—H1*N*1⋯N3^ii^	0.91 (2)	2.28 (2)	3.1773 (19)	168 (2)
C11—H11*A*⋯*Cg*3^iii^	0.95	2.87	3.7667 (17)	158
C19—H19*C*⋯*Cg*2^iv^	0.98	2.69	3.5156 (17)	142

Symmetry codes: (i) 
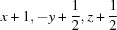
; (ii) 
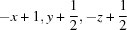
; (iii) 
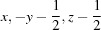
; (iv) 
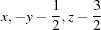
.

**Fig. 1.**
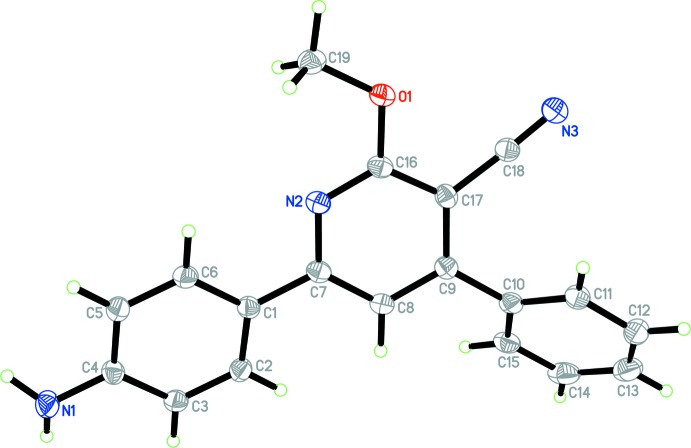
The molecular structure of the title compound, showing 50% probability displacement ellipsoids and the atom-numbering scheme.

**Fig. 2.**
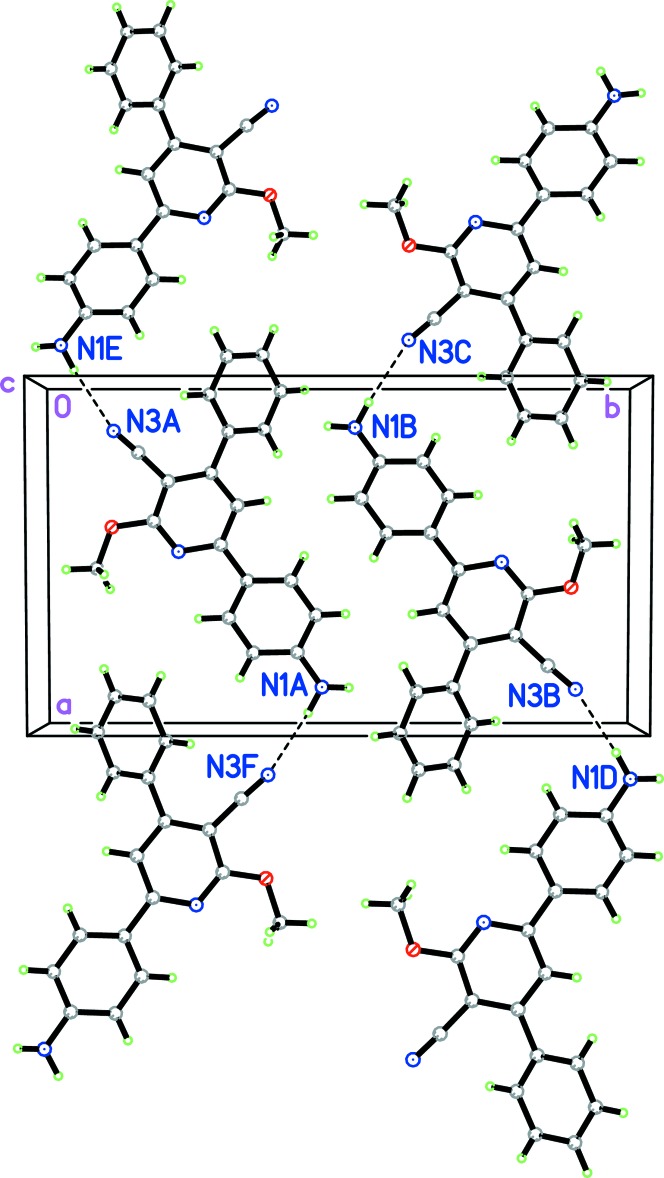
The crystal packing of the title compound viewed approximately along the c axis. N—H···N hydrogen bonds are shown as dashed lines.

**Fig. 3.**
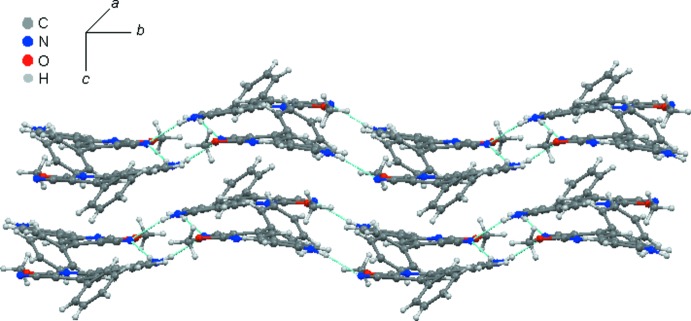
Wave-like sheets of the title compound viewed approximately along the a axis. N—H···N hydrogen bonds are shown as dashed lines.

## supplementary crystallographic information

### 1. Comment

Nicotinonitrile derivatives are substituted pyridines synthesized from the condensation of α,β-unsaturated ketones with malononitrile (Al-Jaber *et al.*, 2012; Zhou *et al.*, 2006). They have a wide range of bioactivities including antitumor, antimicrobial, analgesic, anti-hyperglycemic and antiproliferative activities (Brandt *et al.*, 2010; El-Sayed *et al.*, 2011; Ji *et al.*, 2007; Kim *et al.*, 2005). Nicotinonitriles also find applications as non-linear optical (Raghukumar *et al.*, 2003) and fluorescent materials (Koner *et al.*, 2012). Continuing our ongoing research on fluorescent materials (Chantrapromma *et al.*, 2013; Suwunwong *et al.*, 2012; 2013), the title compound (I) was synthesized and its fluorescent properties were studied. Our results found that (I) possesses significant fluorescent properties that will be discussed elsewhere, together with those of other closely related compounds. Herein the crystal structure of (I) is reported.

The title compound (I), C_19_H_15_N_3_O, is a non-planar molecule (Fig. 1). The pyridine ring makes dihedral angles of 11.50 (7)° and 43.36 (8)° with the 4-aminophenyl and phenyl rings, respectively, and the dihedral angle between the two phenyl rings is 36.28°. The methoxy group lies in the plane of the pyridine ring with an rms deviation of 0.0102 (1) Å for the eight non-H atoms (C7–C9/C16–C17/C19/N2/O1) and the torsion angle C19–O1–C16–N2 = -1.2 (2)°. The cyano group is also roughly co-planar with the pyridine ring with an rms deviation of 0.0406 (1) Å from the ring plane. The bond distances in (I) agree with the literature values (Allen *et al.*, 1987) and are comparable to those found in closely related structures (Chantrapromma *et al.*, 2013 and Suwunwong *et al.*, 2012; 2013).

In the crystal structure (Fig. 2), molecules are linked by intermolecular N—H···N hydrogen bonds (Table 1) forming screw chains. These chains are further linked by N—H···N hydrogen bonds into a two dimensional structure as wave-like sheets parallel to the (1 0 - 2) plane (Fig. 3). These sheets are stacked by π···π interactions between 4-aminophenyl rings of the adjacent sheets with *Cg*_1_···*Cg*_1_^iii, iv^ distances of 3.7499 (9) Å; *Cg*_1_ is the centroid of the N2/C7–C9/C16–C17 pyridine ring. The crystal is further stabilized by C–H···π interactions (Table 1).

### 2. Experimental

The title compound (I) was synthesized by stirring a solution of (*E*)-1-(4-aminophenyl)-3-phenylprop-2-en-1-one (0.22 g, 1 mmol) in methanol (10 ml) with freshly prepared sodium methoxide (1.0 mmol of sodium in 20 ml of methanol). An excess of malononitrile (0.13 g, 2 mmol) was then added with continuous stirring at room temperature until a precipitate was obtained. The resulting solid was filtered. Yellow plate-shaped single crystals of the title compound suitable for *X*-ray structure determination was recrystallized from ethanol/methanol (1:1 *v*/*v*) by slow evaporation of the solvent at room temperature over several days. Mp. 475–476 K.

### 3. Refinement

The amino H atoms were located from difference maps and refined isotropically. The remaining H atoms were positioned geometrically and allowed to ride on their parent atoms, with d(C—H) = 0.95 Å for aromatic and 0.98 Å for CH_3_ atoms. The *U*_iso_ values were constrained to be 1.5*U*_eq_ of the carrier atom for methyl H atoms and 1.2*U*_eq_ for the remaining H atoms. A rotating group model was used for the methyl groups.

### Crystal data

**Table:**

C_19_H_15_N_3_O	*F*(000) = 632
*M**_r_* = 301.34	*D*_x_ = 1.295 Mg m^−^^3^
Monoclinic, *P*2_1_/*c*	Melting point = 475–476 K
Hall symbol: -P 2ybc	Mo *K*α radiation, λ = 0.71073 Å
*a* = 10.9448 (12) Å	Cell parameters from 3696 reflections
*b* = 18.960 (2) Å	θ = 2.2–28.0°
*c* = 7.4738 (8) Å	µ = 0.08 mm^−^^1^
β = 94.743 (2)°	*T* = 100 K
*V* = 1545.6 (3) Å^3^	Plate, yellow
*Z* = 4	0.56 × 0.17 × 0.06 mm

### Data collection

**Table:**

Bruker APEX DUO CCD area-detector diffractometer	3696 independent reflections
Radiation source: sealed tube	2689 reflections with *I* > 2σ(*I*)
Graphite monochromator	*R*_int_ = 0.051
φ and ω scans	θ_max_ = 28.0°, θ_min_ = 2.2°
Absorption correction: multi-scan (*SADABS*; Bruker, 2009)	*h* = −14→14
*T*_min_ = 0.955, *T*_max_ = 0.995	*k* = −24→25
14200 measured reflections	*l* = −9→9

### Refinement

**Table:**

Refinement on *F*^2^	Primary atom site location: structure-invariant direct methods
Least-squares matrix: full	Secondary atom site location: difference Fourier map
*R*[*F*^2^ > 2σ(*F*^2^)] = 0.046	Hydrogen site location: inferred from neighbouring sites
*wR*(*F*^2^) = 0.143	H atoms treated by a mixture of independent and constrained refinement
*S* = 1.07	*w* = 1/[σ^2^(*F*_o_^2^) + (0.0815*P*)^2^ + 0.0098*P*] where *P* = (*F*_o_^2^ + 2*F*_c_^2^)/3
3696 reflections	(Δ/σ)_max_ = 0.001
217 parameters	Δρ_max_ = 0.30 e Å^−^^3^
0 restraints	Δρ_min_ = −0.28 e Å^−^^3^

### Special details

**Table:**

Experimental. The crystal was placed in the cold stream of an Oxford Cryosystems Cobra open-flow nitrogen cryostat (Cosier & Glazer, 1986) operating at 100.0 (1) K.
Geometry. All e.s.d.'s (except the e.s.d. in the dihedral angle between two l.s. planes) are estimated using the full covariance matrix. The cell e.s.d.'s are taken into account individually in the estimation of e.s.d.'s in distances, angles and torsion angles; correlations between e.s.d.'s in cell parameters are only used when they are defined by crystal symmetry. An approximate (isotropic) treatment of cell e.s.d.'s is used for estimating e.s.d.'s involving l.s. planes.
Refinement. Refinement of *F*^2^ against ALL reflections. The weighted *R*-factor *wR* and goodness of fit *S* are based on *F*^2^, conventional *R*-factors *R* are based on *F*, with *F* set to zero for negative *F*^2^. The threshold expression of *F*^2^ > σ(*F*^2^) is used only for calculating *R*-factors(gt) *etc*. and is not relevant to the choice of reflections for refinement. *R*-factors based on *F*^2^ are statistically about twice as large as those based on *F*, and *R*- factors based on ALL data will be even larger.

### Fractional atomic coordinates and isotropic or equivalent isotropic displacement parameters (Å^2^)

**Table:**

	*x*	*y*	*z*	*U*_iso_*/*U*_eq_	
O1	0.41334 (9)	0.11886 (5)	0.32484 (15)	0.0232 (3)	
N1	0.87443 (12)	0.47258 (7)	0.5990 (2)	0.0275 (3)	
H2N1	0.9480 (17)	0.4498 (10)	0.619 (3)	0.036 (5)*	
H1N1	0.8776 (17)	0.5170 (11)	0.554 (3)	0.043 (6)*	
N2	0.48483 (11)	0.23181 (6)	0.37739 (17)	0.0197 (3)	
N3	0.12753 (11)	0.11690 (6)	0.1173 (2)	0.0262 (3)	
C1	0.56914 (12)	0.34757 (7)	0.4295 (2)	0.0188 (3)	
C2	0.56860 (13)	0.42059 (7)	0.4009 (2)	0.0211 (3)	
H2A	0.4974	0.4421	0.3435	0.025*	
C3	0.66877 (13)	0.46216 (7)	0.4540 (2)	0.0222 (3)	
H3A	0.6659	0.5115	0.4320	0.027*	
C4	0.77484 (13)	0.43173 (7)	0.5405 (2)	0.0211 (3)	
C5	0.77592 (13)	0.35885 (8)	0.5715 (2)	0.0231 (3)	
H5A	0.8465	0.3374	0.6310	0.028*	
C6	0.67552 (13)	0.31784 (7)	0.5163 (2)	0.0218 (3)	
H6A	0.6785	0.2684	0.5375	0.026*	
C7	0.46428 (12)	0.30246 (7)	0.3692 (2)	0.0191 (3)	
C8	0.34949 (13)	0.32942 (7)	0.3085 (2)	0.0193 (3)	
H8A	0.3365	0.3790	0.3080	0.023*	
C9	0.25383 (12)	0.28457 (7)	0.2485 (2)	0.0188 (3)	
C10	0.13113 (13)	0.31369 (7)	0.1898 (2)	0.0205 (3)	
C11	0.02459 (13)	0.28273 (8)	0.2451 (2)	0.0245 (3)	
H11A	0.0301	0.2420	0.3193	0.029*	
C12	−0.08913 (14)	0.31153 (9)	0.1918 (2)	0.0315 (4)	
H12A	−0.1613	0.2909	0.2313	0.038*	
C13	−0.09812 (16)	0.37035 (9)	0.0810 (3)	0.0366 (5)	
H13A	−0.1763	0.3894	0.0432	0.044*	
C14	0.00695 (16)	0.40117 (8)	0.0257 (3)	0.0336 (4)	
H14A	0.0008	0.4413	−0.0506	0.040*	
C15	0.12153 (14)	0.37363 (8)	0.0814 (2)	0.0253 (4)	
H15A	0.1935	0.3957	0.0455	0.030*	
C16	0.39525 (12)	0.18928 (7)	0.3197 (2)	0.0192 (3)	
C17	0.27805 (12)	0.21180 (7)	0.2504 (2)	0.0196 (3)	
C18	0.19270 (13)	0.16011 (7)	0.1769 (2)	0.0207 (3)	
C19	0.53384 (14)	0.09527 (8)	0.3919 (2)	0.0253 (4)	
H19A	0.5373	0.0437	0.3870	0.038*	
H19B	0.5509	0.1109	0.5164	0.038*	
H19C	0.5952	0.1152	0.3179	0.038*	

### Atomic displacement parameters (Å^2^)

**Table:**

	*U*^11^	*U*^22^	*U*^33^	*U*^12^	*U*^13^	*U*^23^
O1	0.0235 (5)	0.0159 (5)	0.0299 (7)	−0.0003 (4)	−0.0009 (5)	−0.0005 (4)
N1	0.0209 (7)	0.0207 (6)	0.0401 (9)	−0.0028 (5)	−0.0015 (6)	0.0034 (6)
N2	0.0220 (6)	0.0173 (6)	0.0195 (7)	−0.0007 (4)	0.0010 (5)	−0.0008 (5)
N3	0.0258 (7)	0.0226 (6)	0.0298 (8)	−0.0015 (5)	0.0006 (6)	−0.0012 (6)
C1	0.0190 (7)	0.0201 (7)	0.0172 (8)	−0.0004 (5)	0.0018 (6)	−0.0022 (6)
C2	0.0190 (7)	0.0212 (7)	0.0231 (8)	0.0017 (5)	0.0009 (6)	0.0008 (6)
C3	0.0223 (7)	0.0166 (6)	0.0277 (9)	0.0001 (5)	0.0020 (6)	0.0003 (6)
C4	0.0192 (7)	0.0213 (7)	0.0230 (8)	−0.0025 (5)	0.0033 (6)	−0.0010 (6)
C5	0.0197 (7)	0.0207 (7)	0.0285 (9)	0.0004 (5)	−0.0010 (6)	0.0004 (6)
C6	0.0226 (7)	0.0169 (6)	0.0260 (9)	0.0004 (5)	0.0023 (6)	0.0006 (6)
C7	0.0231 (7)	0.0183 (6)	0.0161 (8)	−0.0008 (5)	0.0029 (6)	−0.0010 (6)
C8	0.0225 (7)	0.0163 (6)	0.0190 (8)	0.0000 (5)	0.0017 (6)	−0.0013 (6)
C9	0.0208 (7)	0.0202 (7)	0.0155 (8)	−0.0011 (5)	0.0028 (5)	−0.0011 (6)
C10	0.0225 (7)	0.0214 (7)	0.0169 (8)	0.0010 (5)	−0.0021 (6)	−0.0056 (6)
C11	0.0241 (7)	0.0260 (7)	0.0233 (9)	−0.0008 (6)	0.0010 (6)	−0.0077 (7)
C12	0.0229 (8)	0.0363 (8)	0.0348 (10)	0.0010 (6)	−0.0002 (7)	−0.0150 (8)
C13	0.0314 (9)	0.0347 (9)	0.0414 (11)	0.0114 (7)	−0.0105 (8)	−0.0154 (8)
C14	0.0424 (9)	0.0238 (7)	0.0323 (10)	0.0099 (7)	−0.0101 (8)	−0.0056 (7)
C15	0.0316 (8)	0.0200 (7)	0.0235 (9)	0.0010 (6)	−0.0023 (7)	−0.0052 (6)
C16	0.0240 (7)	0.0163 (6)	0.0177 (8)	−0.0014 (5)	0.0041 (6)	0.0006 (6)
C17	0.0197 (7)	0.0212 (7)	0.0179 (8)	−0.0025 (5)	0.0015 (6)	−0.0011 (6)
C18	0.0224 (7)	0.0194 (6)	0.0201 (8)	0.0001 (5)	0.0016 (6)	0.0005 (6)
C19	0.0280 (8)	0.0199 (7)	0.0277 (9)	0.0030 (6)	0.0004 (6)	0.0026 (6)

### Geometric parameters (Å, º)

**Table:**

O1—C16	1.3498 (16)	C8—C9	1.3937 (18)
O1—C19	1.4427 (17)	C8—H8A	0.9500
N1—C4	1.3786 (18)	C9—C17	1.405 (2)
N1—H2N1	0.916 (19)	C9—C10	1.4844 (18)
N1—H1N1	0.91 (2)	C10—C15	1.395 (2)
N2—C16	1.3144 (17)	C10—C11	1.398 (2)
N2—C7	1.3589 (17)	C11—C12	1.387 (2)
N3—C18	1.1513 (18)	C11—H11A	0.9500
C1—C2	1.401 (2)	C12—C13	1.388 (3)
C1—C6	1.4032 (18)	C12—H12A	0.9500
C1—C7	1.4718 (18)	C13—C14	1.383 (3)
C2—C3	1.3815 (19)	C13—H13A	0.9500
C2—H2A	0.9500	C14—C15	1.390 (2)
C3—C4	1.4052 (19)	C14—H14A	0.9500
C3—H3A	0.9500	C15—H15A	0.9500
C4—C5	1.401 (2)	C16—C17	1.4094 (19)
C5—C6	1.3811 (19)	C17—C18	1.4317 (19)
C5—H5A	0.9500	C19—H19A	0.9800
C6—H6A	0.9500	C19—H19B	0.9800
C7—C8	1.3968 (19)	C19—H19C	0.9800
			
C16—O1—C19	116.38 (10)	C15—C10—C11	119.30 (13)
C4—N1—H2N1	116.6 (11)	C15—C10—C9	119.85 (13)
C4—N1—H1N1	117.2 (12)	C11—C10—C9	120.84 (14)
H2N1—N1—H1N1	116.0 (17)	C12—C11—C10	120.01 (15)
C16—N2—C7	118.28 (12)	C12—C11—H11A	120.0
C2—C1—C6	117.41 (12)	C10—C11—H11A	120.0
C2—C1—C7	122.29 (12)	C11—C12—C13	120.37 (16)
C6—C1—C7	120.29 (12)	C11—C12—H12A	119.8
C3—C2—C1	121.80 (13)	C13—C12—H12A	119.8
C3—C2—H2A	119.1	C14—C13—C12	119.87 (15)
C1—C2—H2A	119.1	C14—C13—H13A	120.1
C2—C3—C4	120.24 (13)	C12—C13—H13A	120.1
C2—C3—H3A	119.9	C13—C14—C15	120.24 (16)
C4—C3—H3A	119.9	C13—C14—H14A	119.9
N1—C4—C5	120.41 (13)	C15—C14—H14A	119.9
N1—C4—C3	121.11 (13)	C14—C15—C10	120.19 (16)
C5—C4—C3	118.45 (12)	C14—C15—H15A	119.9
C6—C5—C4	120.69 (13)	C10—C15—H15A	119.9
C6—C5—H5A	119.7	N2—C16—O1	119.59 (12)
C4—C5—H5A	119.7	N2—C16—C17	124.49 (13)
C5—C6—C1	121.41 (13)	O1—C16—C17	115.92 (12)
C5—C6—H6A	119.3	C9—C17—C16	117.80 (12)
C1—C6—H6A	119.3	C9—C17—C18	123.51 (12)
N2—C7—C8	121.10 (12)	C16—C17—C18	118.60 (13)
N2—C7—C1	115.91 (12)	N3—C18—C17	177.54 (15)
C8—C7—C1	122.99 (12)	O1—C19—H19A	109.5
C9—C8—C7	120.85 (12)	O1—C19—H19B	109.5
C9—C8—H8A	119.6	H19A—C19—H19B	109.5
C7—C8—H8A	119.6	O1—C19—H19C	109.5
C8—C9—C17	117.39 (12)	H19A—C19—H19C	109.5
C8—C9—C10	120.36 (12)	H19B—C19—H19C	109.5
C17—C9—C10	122.24 (12)		
			
C6—C1—C2—C3	0.6 (2)	C8—C9—C10—C11	−135.09 (16)
C7—C1—C2—C3	−178.29 (15)	C17—C9—C10—C11	43.4 (2)
C1—C2—C3—C4	−0.5 (2)	C15—C10—C11—C12	0.2 (2)
C2—C3—C4—N1	−178.14 (15)	C9—C10—C11—C12	178.71 (14)
C2—C3—C4—C5	−0.2 (2)	C10—C11—C12—C13	1.1 (2)
N1—C4—C5—C6	178.73 (16)	C11—C12—C13—C14	−1.0 (3)
C3—C4—C5—C6	0.7 (2)	C12—C13—C14—C15	−0.3 (3)
C4—C5—C6—C1	−0.7 (2)	C13—C14—C15—C10	1.6 (3)
C2—C1—C6—C5	0.0 (2)	C11—C10—C15—C14	−1.5 (2)
C7—C1—C6—C5	178.90 (15)	C9—C10—C15—C14	179.94 (14)
C16—N2—C7—C8	2.8 (2)	C7—N2—C16—O1	179.46 (13)
C16—N2—C7—C1	−177.88 (13)	C7—N2—C16—C17	−0.5 (2)
C2—C1—C7—N2	169.00 (15)	C19—O1—C16—N2	−1.2 (2)
C6—C1—C7—N2	−9.9 (2)	C19—O1—C16—C17	178.69 (14)
C2—C1—C7—C8	−11.7 (2)	C8—C9—C17—C16	2.7 (2)
C6—C1—C7—C8	169.48 (15)	C10—C9—C17—C16	−175.87 (14)
N2—C7—C8—C9	−2.3 (2)	C8—C9—C17—C18	−173.75 (14)
C1—C7—C8—C9	178.41 (14)	C10—C9—C17—C18	7.7 (2)
C7—C8—C9—C17	−0.5 (2)	N2—C16—C17—C9	−2.3 (2)
C7—C8—C9—C10	178.05 (14)	O1—C16—C17—C9	177.76 (14)
C8—C9—C10—C15	43.4 (2)	N2—C16—C17—C18	174.30 (15)
C17—C9—C10—C15	−138.05 (15)	O1—C16—C17—C18	−5.6 (2)

### Hydrogen-bond geometry (Å, º)

*Cg*2 and *Cg*3 are the centroids of the C1–C6 and C10–C15 rings, respectively.

**Table:**

*D*—H···*A*	*D*—H	H···*A*	*D*···*A*	*D*—H···*A*
N1—H2*N*1···N3^i^	0.915 (19)	2.338 (19)	3.2416 (18)	169.5 (19)
N1—H1*N*1···N3^ii^	0.91 (2)	2.28 (2)	3.1773 (19)	168 (2)
C11—H11*A*···*Cg*3^iii^	0.95	2.87	3.7667 (17)	158
C19—H19*C*···*Cg*2^iv^	0.98	2.69	3.5156 (17)	142

Symmetry codes: (i) *x*+1, −*y*+1/2, *z*+1/2; (ii) −*x*+1, *y*+1/2, −*z*+1/2; (iii) *x*, −*y*−1/2, *z*−1/2; (iv) *x*, −*y*−1/2, *z*−3/2.
